# Ablation of Atrial Tachycardia in a Budd‐Chiari Syndrome Patient via Inferior Vena Cava–Azygos–Superior Vena Cava Approach

**DOI:** 10.1111/anec.70136

**Published:** 2025-12-17

**Authors:** Yuwei Chen, Yi Liu, Xiangbin Xiao, Xiaobo Pu

**Affiliations:** ^1^ Department of Cardiovascular Medicine The Affiliated Hospital of Southwest Medical University Luzhou Sichuan China; ^2^ Department of Cardiovascular Medicine The People's Hospital of Jianyang City Jianyang China; ^3^ Department of Cardiology West China Hospital, Sichuan University Chengdu China

**Keywords:** 3D electroanatomical mapping, atrial tachycardia ablation, Budd‐Chiari syndrome, catheter ablation, IVC‐azygos‐SVC approach

## Abstract

**Introduction:**

Catheter ablation for atrial tachycardia (AT) in Budd‐Chiari syndrome (BCS) presents unique challenges due to altered venous anatomy. This case demonstrates an innovative approach to overcome complete inferior vena cava (IVC) occlusion.

**Methods:**

A 24‐year‐old female with BCS underwent catheter ablation via an innovative femoral vein–IVC–azygos–SVC approach, guided by preprocedural CT angiography and 3D electroanatomical mapping.

**Results:**

Successful ablation was achieved at the anterior interatrial septum with no arrhythmia recurrence during the 3‐month follow‐up.

**Conclusion:**

This case demonstrates the successful use of an IVC‐azygos‐SVC approach guided by CT angiography and 3D mapping for atrial tachycardia ablation in Budd‐Chiari syndrome, offering a viable solution for patients with complex venous obstruction.

## Abstract 2

This study demonstrates a successful catheter ablation for atrial tachycardia in Budd‐Chiari syndrome via an innovative IVC‐azygos‐SVC approach, overcoming complete IVC occlusion. Preprocedural CT angiography and 3D mapping were critical to navigating the altered anatomy, offering a safe alternative for patients with complex venous obstruction.

## 1 Introduction

Budd‐Chiari syndrome (BCS) is a rare but clinically significant disorder characterized by hepatic venous outflow obstruction, frequently resulting in extensive collateral circulation. While catheter ablation is a well‐established treatment for atrial tachycardia (AT), patients with BCS present unique anatomical challenges that complicate standard interventional strategies. This case report discusses a 24‐year‐old female with BCS and recurrent AT, where conventional ablation access was impossible due to complete IVC occlusion. The case highlights the critical importance of comprehensive preprocedural imaging and advanced mapping techniques in overcoming such anatomical barriers, while demonstrating the viability of an inferior vena cava–azygos–superior vena cava approach for successful catheter ablation.

## 2 Case

**FIGURE 1:**
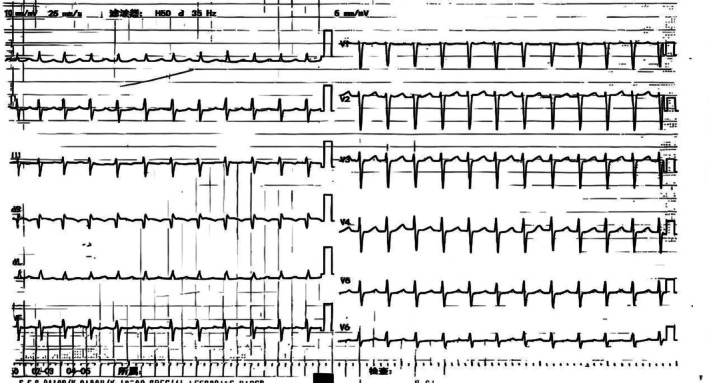
Twelve‐lead electrocardiogram indicated a narrow QRS complex tachycardia.

**FIGURE 2:**
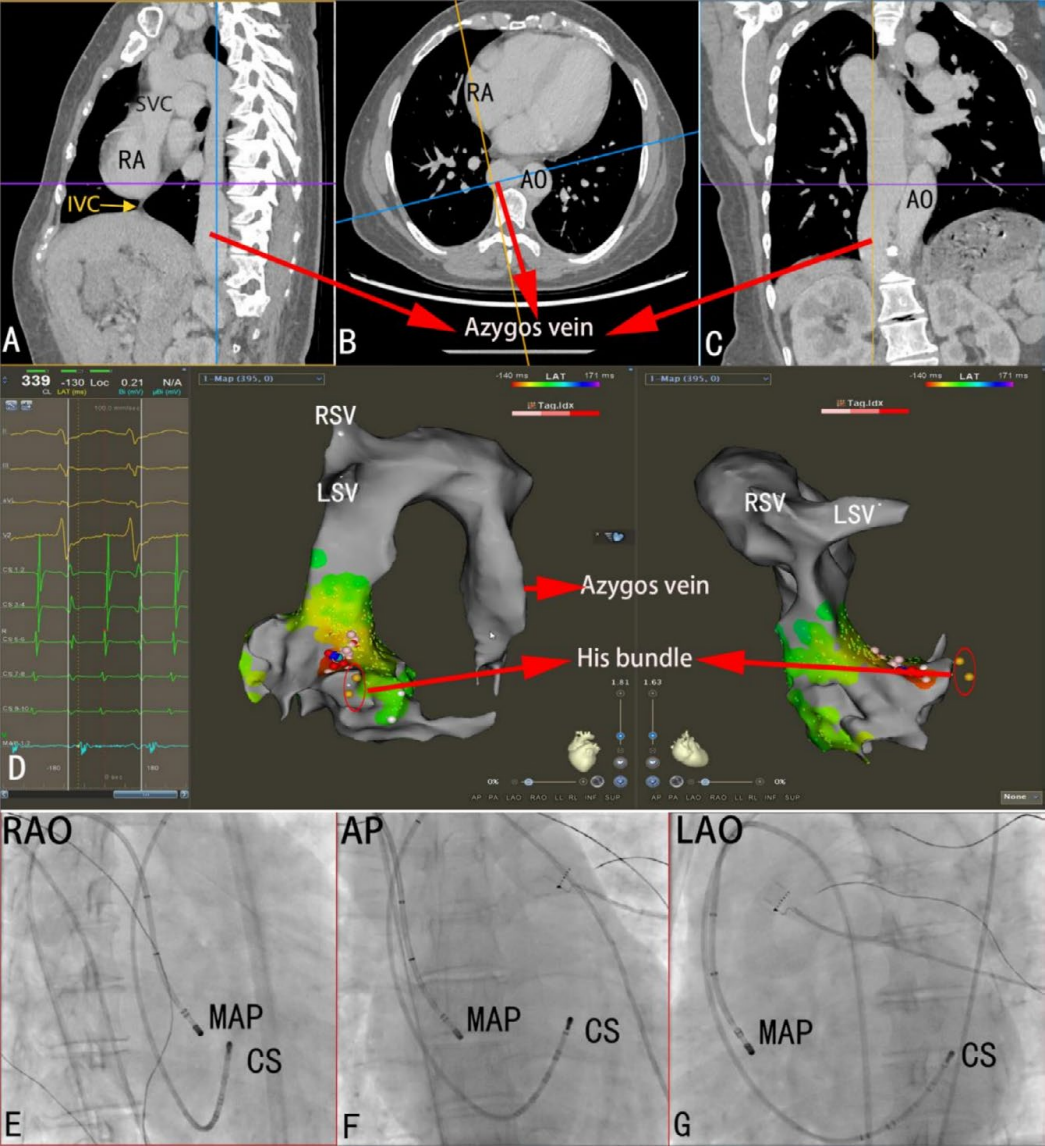
(A–C) Preoperative CTA demonstrating IVC occlusion (yellow arrow) and collateral drainage via azygos vein. (D) 3D electroanatomical map highlighting earliest activation site (blue dot) at anterior interatrial septum. (E–G) Fluoroscopic images confirming catheter positions (CS and mapping catheters) during ablation.

A 24‐year‐old female was admitted for recurrent paroxysmal palpitations. A twelve‐lead electrocardiogram indicated a narrow QRS complex tachycardia (Figure 1). She had been diagnosed with Budd‐Chiari syndrome (BCS) and received percutaneous transluminal angioplasty of the inferior vena cava (IVC) and hepatic vein 4 years ago. Preoperative computed tomography angiography (CTA, Figure 2A–C) confirmed that the hepatic segment of the IVC (yellow arrow) was occluded, and the blood flow from the IVC was drained through the significantly dilated azygos vein, onward to the superior vena cava (SVC), and finally to the right atrium (RA), indicating that femoral vein‐ICV‐azygos‐SVC may be the feasible approach for ablation of the tachycardia. A decapolar catheter was inserted into the coronary sinus from the left subclavian vein (LSV), and an ablation catheter was passed into the right atrium via the femoral vein‐ICV‐azygos‐SVC approach. Meanwhile, a three‐dimensional (3D) electroanatomical image was reconstructed from the azygos to the RA by an ablation catheter with a force monitor. Intravenous isoproterenol infusion was administered to elevate the heart rate, followed by atrial S1S1‐burst stimulation, which induced a narrow QRS complex tachycardia at a rate of 150 beats per minute. Activation mapping demonstrated that the earliest site was located at the anterior interatrial septum (blue dot), and tachycardia was eliminated at the focal firing (Figure 2D). Fluoroscopy confirmed the position of mapping (Map) and the coronary sinus (CS) catheter (Figure 2E–G). No recurrence of any form of tachycardia was recorded 3 months later.

## 3 Discussion

Budd‐Chiari syndrome is an uncommon disease in the West; the estimated incidence is one in 2.5 million per person‐year (Zu et al. 2020). Research shows that the IVC obstruction is most common in Asia; collateral pathways like the azygous may take over the venous drainage as compensation, which provides us with a possible approach for ablation of tachycardia (Hu et al. 2016; Piotr 2018).

This case highlights the importance of identifying abnormal anatomy before the procedure through detailed structure analysis using multiplanar reformation CTA. In addition, the application of 3D electroanatomical mapping can reconstruct the image of abnormal anatomy, which provides us with an intuitive view. In patients with BCS, due to the occlusion of the inferior vena cava, the conventional approach is not feasible. A thorough understanding of the patient's past medical history before the operation, in‐depth analysis of the compensatory pathways of inferior vena cava return through vascular CT imaging, and the combination of 3D electroanatomical mapping to clearly display the feasible vascular access are the reasons and prerequisite conditions for the successful intracardiac electrophysiological examination and catheter radiofrequency ablation.

## 4 Conclusion

This case demonstrates the feasibility of catheter ablation for atrial tachycardia in Budd‐Chiari syndrome via an IVC‐azygos‐SVC approach when standard venous access is obstructed. The successful outcome highlights the critical role of preprocedural CT angiography and 3D electroanatomical mapping in navigating complex venous anatomy.

## Author Contributions

**Yuwei Chen:** data curation, investigation, writing – original draft. **Yi Liu:** formal analysis, investigation, writing – review and editing. **Xiaobo Pu:** resources, supervision, validation. **Xiangbin Xiao:** conceptualization, methodology, project administration, supervision, writing – review and editing.

## Funding

The authors have nothing to report.

## Ethics Statement

This case report is based on a surgical intervention that was performed as part of the routine clinical practice and quality improvement. Therefore, ethical approval from an institutional review board or ethics committee is not required for this report. The patient gave his written consent for the intervention and for the publication of this case report.

## Conflicts of Interest

The authors declare no conflicts of interest.

## Data Availability

No datasets were generated or analyzed during the current study.
